# EVENS (Evaluation Nursing Students): A Mobile Application to Enhance Nursing Students’ Clinical Competence and Self-Efficacy—A Quasi-Experimental Study

**DOI:** 10.3390/nursrep16030083

**Published:** 2026-02-27

**Authors:** María Isabel Guzmán-Almagro, Rosa M. Carro, Pablo Izaguirre-García, Francisco Félix Caballero-Díaz, Miriam Leñero-Cirujano, Cristina Oter-Quintana, María Teresa González-Gil, María Teresa Alcolea-Cosín, Carmen García-García, Ana Isabel Parro-Moreno

**Affiliations:** 1 Department of Nursing, Faculty of Medicine, Autonomous University of Madrid, 28049 Madrid, Spain; isabel.guzman@uam.es (M.I.G.-A.); mteresa.alcolea@uam.es (M.T.A.-C.); 2 La Paz University Hospital, 28046 Madrid, Spain; 3 Department of Computer Engineering, School of Engineering, Autonomous University of Madrid, 28049 Madrid, Spain; rosa.carro@uam.es (R.M.C.); pablo@izaguirre.es (P.I.-G.); 4 Department of Preventive Medicine, Public Health and Microbiology, Faculty of Medicine, Autonomous University of Madrid, 28029 Madrid, Spain; felix.caballero@uam.es; 5 Department of Nursing, Faculty of Nursing, Physiotherapy and Podiatry, Complutense University of Madrid, 28040 Madrid, Spain; mlcirujano@ucm.es; 6 Department of Nursing, Faculty of Medicine, Autonomous University of Madrid, IDIPHISA (Instituto de Investigación Sanitaria Puerta de Hierro-Segovia de Arana), 280492 Madrid, Spain; cristina.oter@uam.es (C.O.-Q.); mariat.gonzalez@uam.es (M.T.G.-G.); 7 Department of Social Psychology and Methodology, Faculty of Psychology, Autonomous University of Madrid, 28049 Madrid, Spain; carmen.garcia@uam.es

**Keywords:** nursing education, clinical practicum, educational technologies, mobile application, nurse teacher, nursing student, evaluation, self-efficacy

## Abstract

**Background/Objectives:** Evaluation of students in practicums is essential in their training process. Mobile technologies enable formative assessments in training, enhance feedback, and improve students’ clinical competence and self-efficacy. Nevertheless, in the absence of previous evidence, their effects on clinical learning must be evaluated with rigor and caution. We aimed to evaluate the improvement in nursing students’ clinical competence and self-efficacy during their clinical practicums using the Evaluation Nursing Student (EVENS) application. **Methods**: A quasi-experimental design with non-equivalent control and intervention groups was adopted. Participants were not randomly assigned. The inclusion criterion was enrolment for the Supervised Practicum II course in the Nursing degree course at University X. Students agreeing to use the EVENS application during their Supervised Practicum II were assigned to the intervention group. The primary outcomes were student competence and self-efficacy, and the secondary outcome was the usability of the application. The analysis included a comparison of the pre- and post-intervention means of the intervention and control groups using Student’s *t*-tests. **Results**: One hundred and forty-nine mostly female (*n* = 137, 91.9%) students participated in the study. Forty-eight were assigned to the intervention group and 101 to the control group. No statistically significant differences regarding clinical competence or self-efficacy were found between the groups. Tutors rated the application’s usability with an average of 3.8 out of 5. **Conclusions**: The use of the EVENS application did not improve the primary outcomes. Although it was positively received by tutors as supportive of their role in training students engaged in clinical practicums.

## 1. Introduction

In response to European guidelines, the training of future nurses places special emphasis on practical training credits in real (not simulated) social and healthcare settings. In Spain, the curriculum for the Bachelor’s Degree in Nursing requires students to complete 2550 h of clinical practice at the patient’s bedside to ensure that they acquire the skills associated with the degree [1]. Clinical practice hours are distributed across multiple practicums. These practicums are scheduled throughout the years of training. Their duration in weeks varies, increasing in the later years of the degree, during which students’ placement in clinical settings may exceed 30 weeks.

During clinical practice, nursing students are expected to acquire knowledge, attitudes, and skills through experiential learning. The support of clinical tutors is essential for this to happen. This bedside support must be mediated by fluid communication and constant stimulation of critical reflection with constructive feedback [2].

The nature of healthcare dynamics means that the student is not always accompanied by a single clinical tutor throughout their clinical rotation. This means that many professionals may be involved in supporting the student, often with limited continuity and difficulties in communication regarding the student’s progress and achievements [3,4,5,6].

However, these clinical tutors must communicate with each other during the clinical practice in order to monitor the student’s learning process thoroughly. This issue, combined with other difficulties such as clinicians’ lack of motivation to participate in students’ training, shortcomings in pedagogical knowledge, and excessive workloads, are significant barriers and stressors that hinder the learning process of nursing students.

Several studies have shown that the integration of new technologies can enhance the teaching–learning process by facilitating the acquisition of specific skills [4], fostering critical thinking [7], enhancing self-efficacy, and improving student satisfaction [8,9,10,11,12,13].

New technologies can also foster communication and cooperation between students and clinical tutors [12], enabling closer interaction, a sense of connectedness, and more continuous and immediate feedback [14].

The use of hand-held mobile and electronic devices such as smartphones, tablets, and personal computers is readily accepted by young people and may prove to be an accessible and viable resource for students, providing easy access to information and feedback about their clinical practicums.

At University X, clinical placements for nursing degree students begin in the second year, and the assessment of clinical competencies includes an evaluation by the clinical tutor. This assessment is problematic due to its summative and final nature, and feedback for students on their clinical competence is limited to the time they spend in clinical settings. This hinders formative assessment and may affect students’ self-efficacy.

**Figure 1:**
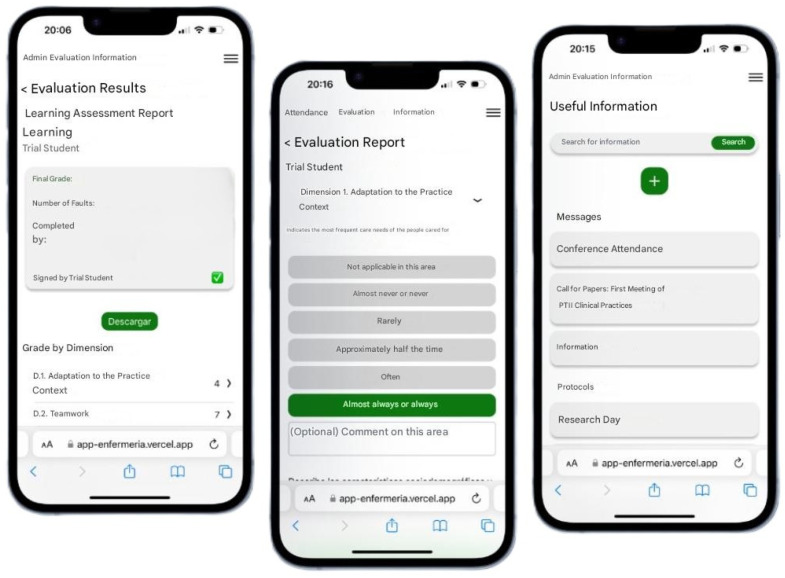
Application screenshots displaying information about clinical practicum data.

The Evaluation Nursing students (EVENS) [software, cited 25 March 2026, avaible from https://app-enfermeria.vercel.app/auth/signup])application was developed in collaboration with the Department of Computer Engineering at (University X) in order to overcome this problem. The web-enabled software can be used on smartphones, tablets, and PCs, and monitors the competence level attained by students during their practicum using tutors’ dynamic records of qualitative and quantitative annotations. This data are presented in graph format, as well as through instant messaging and access to documents and relevant links containing clinical data related to the practicum (Figure 1).

Given that the app was designed to support formative assessment, it was hypothesized that its use would enhance students’ clinical competence and self-efficacy. The study aims to help address the lack of evidence regarding the impact of technology—particularly mobile applications—on clinical learning as pointed out in the meta-analysis conducted by Chen et al. [13].

STUDY AIM:

To evaluate the improvement in nursing students’ clinical competence and self-efficacy during their clinical practicums using the Evaluation Nursing Student (EVENS) application.

## 2. Materials and Methods

DESIGN: A quasi-experimental design with non-equivalent control and intervention groups was adopted. Participants were not randomly assigned to the groups, and the intervention group comprised those students who enrolled voluntarily to the EVENS app during their practical training.

SUBJECTS AND SCOPE OF THE STUDY

The study population consisted of all the students enrolled in the Supervised Practicum II course in their second year of nursing studies at Departament of nursing (Autonomus University of Madrid, Faculty of Medicine, Spain). The clinical rotation lasted four weeks in medical–surgical units at three public hospitals and 32 health centers within the Madrid healthcare service, with students completing seven hours of clinical practice per day, five days per week. All the students had previously completed the first rotation, which also lasted four weeks.

**The inclusion criterion** for the study population was enrolment for the Supervised Practicum II course for the first time.

Forty-eight of the students enrolled (149) agreed to use EVENS during their practical training. These students were assigned to the intervention group. The remaining 101 students who did not agree were assigned to the control group.

The 48 clinical tutors who accompanied the 48 students in the intervention group during the four weeks of clinical practice were also included in the study. They all agreed to participate by monitoring the students using the EVENS app. The study was conducted from January to May 2024.

SELECTION OF PARTICIPANTS:

All the students (potential participants) were informed about the teaching-research innovation project in a formal session scheduled within the academic calendar. This oral information was also reinforced with written information that was made available to students to read carefully on the digital educational platform for the Supervised Practicum II course. The students were given the email addresses of the project coordinator and course coordinator so that they could ask any questions about the project and their participation in it. Participation in the study was voluntary. All the participating students signed an informed consent form (as explained in more detail in the Ethical Considerations section).

Control Group:

The students in the control group were accompanied and assessed according to the standard procedure, as set out in the teaching guide and using the instruments provided. These are: direct oral communication in interaction spaces agreed between the student and the clinical tutor (mentoring spaces), and using forms validated by the Nursing Department (in the form of a checklist with spaces for observations) covering the various competencies to be acquired in the practical course, broken down into indicators.

Two formal feedback sessions are conducted using this form during the rotation: one halfway through it (in the second or third week) and one at the end (at the conclusion of the rotation). These reports are agreed upon with the students and aim to provide constructive feedback focused on the students’ development.

The students who did not wish to participate in the study received their assessment orally and in writing from their assigned clinical tutor in the assessment report at the conclusion of the clinical practicum.

Intervention Group:

The procedure for students who wished to participate in the intervention during their rotation was equivalent to the usual procedure applied to the control group but carried out using EVENS (App) instead of the aforementioned forms.

As a preliminary step, both the students and teachers were informed about its structure and content and trained in its use. This information and training were provided in scheduled meetings and tutorial videos that were made available on the digital education platform. These sessions also reinforced key aspects of formative assessment for experiential learning.

The teaching staff was instructed to use the app at any time to record students’ performance, add notes, make qualitative comments, and engage in additional assessment and monitoring activities. This was intended to create a continuous monitoring process, enabling the clinical tutor and faculty professor to enter information at any time, and to give students access to this information as continuous feedback.

The app included information about the student, tutor, and assigned teacher, unit, clinical practice schedule, learning competencies, other useful information, such as the procedure to follow in the event of a biological accident, and a space for communication between the student, tutor, and teacher, similar to a social network. It also provided a graphical representation of all the assessment inputs by the tutor during the clinical practice period.

The intervention was designed to facilitate cooperation between students and teachers by placing communication procedures in a central digital environment, allowing for flexibility, convenience, and availability, and facilitating synchronous and asynchronous communication regardless of time and place.

The app also provided students with immediate feedback on their learning progress, enabling them to take action if necessary. Teachers were also able to monitor students’ progress more quickly and offer support at the appropriate time. The clinical tutor was the main figure involved in the intervention and received training on the app’s features and the intervention procedures. They were instructed on the importance of providing continuous feedback throughout the rotation, with frequent notes (10 times was the recommended amount) in accordance with the concept of formative assessment.

Best practices related to security were implemented, including user authentication and password encryption. Access to EVENS required registration through a web-based link. All students, clinical tutors, and teachers therefore interacted in a private and secure space.

EVENS was piloted during the year prior to its implementation with a group of 3 clinical tutors and 3 second-year students engaged in clinical practicums. The results were satisfactory in terms of the tutors’ willingness to use the application [15] and served to improve functionalities and overcome the difficulties that arose.

OUTCOME MEASURES/Data collection

The primary outcomes were those related to the competence and self-efficacy achieved by students and were collected for both the intervention and control groups, pre- and post-intervention.

**Figure 2:**
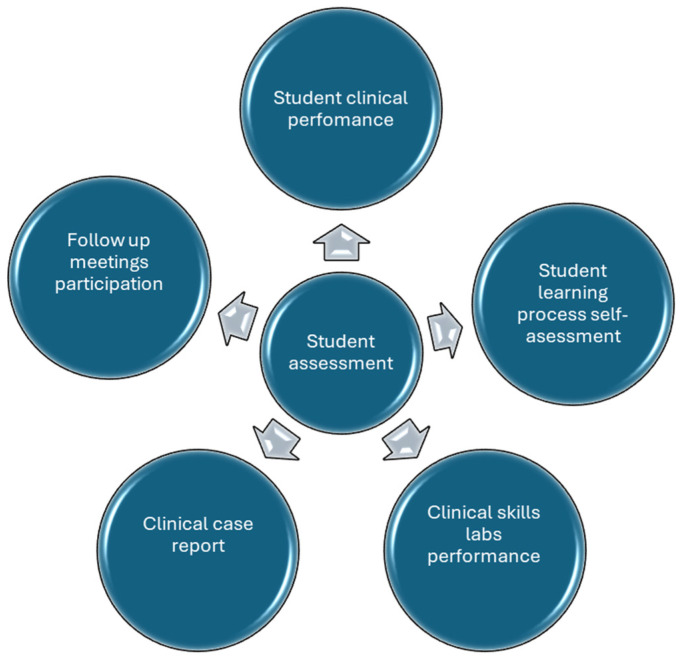
Aspects of student assessment.

The competence data were obtained from various tools usually used to evaluate students in the practicum in the Department of Nursing, including (Figure 2):

*Student clinical performance* (the Clinical Tutor evaluates the learning process and the student’s achievements in terms of competency level acquisition),

*Student learning process self-assessment* (the student self-assesses his/her learning process and achievements in terms of competency level acquisition),

*Clinical skills labs performance* (the Faculty Professor evaluates the student’s performance in clinical skills laboratories taught prior to the clinical internship),

*Clinical case report* (the Faculty Professor evaluates the student’s ability to integrate theoretical knowledge into a case study),

*Follow-up meetings participation* (the Faculty Professor evaluates the student’s participation in and use of follow-up tutoring).

The self-efficacy data were collected using the General Self-Efficacy Scale, validated for the Spanish context [16] with a Cronbach’s alpha of 0.87. This scale aims to assess individuals’ feelings regarding their capacity to cope with everyday stress, with scores ranging between 1 and 10 points for ten items, with the highest possible score being 100 points.

The secondary outcomes were usability and satisfaction with EVENS, based on information provided by the clinical tutors. The usability questionnaire used was the USU, validated for the Spanish context and recommended for use with this type of software [17]. The scores range from 0 to 5, where 5 is the best possible score, for a total of 10 items. This system offers good psychometric properties, with a Cronbach’s alpha of 0.92. The tutors’ satisfaction with EVENS was recorded in the form of qualitative remarks in free text.

DATA COLLECTION

The data were collected between January and May 2024. The “pre” data were collected in January, at the end of the first clinical practicum, and the “post” data in May, at the end of the second clinical practicum. The data relating to student competence and self-efficacy were collected from databases, records, and questionnaires. The usability and satisfaction reported by the tutors were collected from records from the application at the end of the second practicum. The data concerning application activity (the number of times and duration of access to the app by tutors and students alike) were collected at the end of the second practicum.

ETHICAL CONSIDERATIONS

This project was approved by the Ethics Committee of University X as compliant with the ethical requirements for its execution. All the potential participants were previously informed about the aims of the research, the treatment and anonymization of the study results, and that they could withdraw from the project at any time, with no questions asked and no academic repercussions. The participants were required to sign an informed consent document before joining the study. Participation in the project did entail any benefit for students in terms of academic remuneration, and non-participation had no negative effect or penalty.

DATA ANALYSIS

A descriptive analysis was conducted in which the mean scores and percentages were calculated (depending on the continuous or categorical nature of each variable), as well as the standard deviation as a measure of dispersion for the continuous variables.

Mean scores in the intervention and control groups were compared on the basis of the scores obtained in the second semester and by means of Student’s *t*-test for independent samples. Paired Student’s *t*-tests were used for comparing mean pre-intervention and post-intervention scores in the intervention group. Pre-intervention scores corresponded with those obtained in clinical practicums at the end of the first semester (January), while post-intervention scores corresponded with those obtained at the end of second-semester clinical practicums (May).

The confidence level considered in the statistical hypothesis tests was 95%. For the statistical analysis, we used Stata version 17.

## 3. Results

The intervention group comprised 48 students, and there were 101 students in the control group. Their median age was 20 (Min 20; Max 38) years. The majority (*n* = 137, 91.9%) were women.

The highest mean score of 9 (S.D. = 0.9) was consistent with the students’ clinical performance. The mean values for scores related to the level of knowledge were 8.5 ± 1.1 for clinical case reports corrected by the teacher, and 8.5 ± 1 for clinical skills labs. The mean score for students’ self-assessment of their level of competence was 9.1 ± 0.9 (Table 1). Pre-test scores in both intervention and control groups are shown as supplementary material (Supplementary Table S1), without significant differences between the two groups.

No statistically significant differences were identified when comparing the two groups’ results for the different aspects of evaluation. However, lower mean scores than in the control group were observed both for self-assessment in the intervention group (8.9 ± 1.4 vs. 9.2 ± 0.6) and in evaluation by tutors (9.0 ± 0.8 vs. 9.1 ± 0.9). The mean score in the self-efficacy questionnaire was lower in the intervention group (73 ± 11.1 vs. 76.5 ± 16.9), although this difference was not significant (*p* = 0.60).

Although analyses of differences were conducted before and after the intervention in both groups, no statistically significant differences were found except for the scores for clinical case reports, which were better in the second semester for both groups. These ranged from 7.8 ± 1.3 to 8.5 ± 1.1 in the intervention group [t (45) = 2.64, *p* = 0.01], and from 7.9 ± 1.3 to 8.6 ± 1.1 in the control group [t (95) = 3.86, *p* < 0.001]. Likewise, a statistically significant improvement in the mean score for self-assessment from 8.9 ± 0.7 to 9.2 ± 0.6 [t (95) = 4.93, *p* < 0.001] was observed in the control group (Table 2).

**Figure 3:**
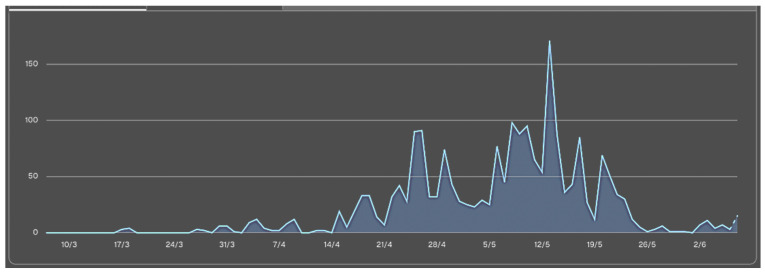
Graph showing activity over time. Y-axis: number of registrations and visits. X-axis: weeks of the rotation (4 weeks in total).

**Figure 4:**
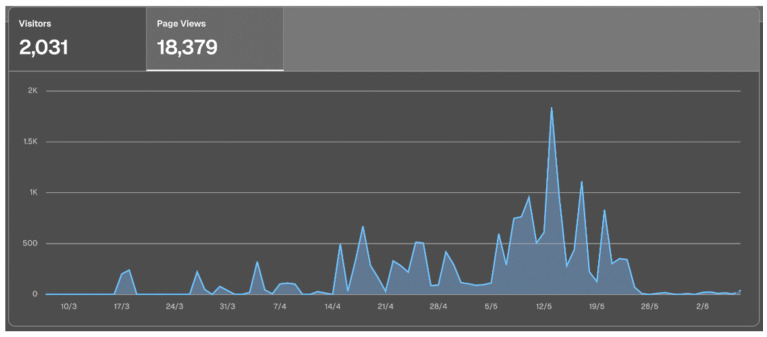
Graph showing the number of views. Y-axis: number of views. X-axis: weeks of the rotation (4 weeks in total).

With regard to application use, both the number of users and the number of different pages used per day gradually increased as the practicum unfolded, reaching a peak of concentrated activity in the final week (Figure 3 and Figure 4).

The usability questionnaire was answered by 42 tutors, with 6 not responding. The highest-scoring items were “I think the website is easy to use”, “I found that several functions were well integrated”, “I think there was consistency on the website”, “I found the website highly intuitive”, and “I was able to use the website without having to learn anything new” (Table 3).

## 4. Discussion

No improvement in learning attributable to the intervention with EVENS was confirmed, as no statistically significant pre- and post- differences were observed in the variables studied. However, improvements were identified from the point of view of learning. The mean scores for academic work in both groups were better, probably as a result of the increase in knowledge and training acquired over time and with clinical experience. The control group also showed an improvement in self-assessment that was not observed in the intervention group. This is an interesting area that should be explored to see how it may be affected by a tool that promotes feedback and should therefore foster students’ self-awareness of their level of competence.

The scores for self-efficacy levels were higher after the intervention in both groups, and also higher than in another study conducted with a sample of Spanish university students using the same questionnaire [16]. It is logical to assume that clinical experience has a positive influence on students’ self-efficacy. Based on our teaching experience, we believe that formative assessment should work to improve this concept.

Low levels of self-efficacy are associated with lower levels of self-esteem, poorer grades, and poorer performance in stressful situations [17]. Better results for self-efficacy are associated with better feelings, thoughts, decision-making, and interpersonal relationships, which are key elements in clinical learning. As teachers, we can and must foster self-efficacy, even during online interventions, given the positive results gained in clinical practicums as a result [9,18].

In the test conducted by Strandell et al. on the impact of an online study on monitoring of students engaged in clinical practicums, statistically significant differences between the two groups in terms of competence, knowledge, and self-efficacy were likewise absent [12]. The authors attributed this to the short duration of the intervention, which lasted just five weeks. The meta-analysis conducted by Sung et al. (2016) [19] on the use of mobile devices in education, points out that although mobile devices may improve education, the true impact of mobile learning programs must be enhanced by interventions with a longer duration. For instance, interventions on research or cooperative learning may require a longer time span to guarantee the outcome of mobile programs.

Regarding the tutor-student feedback process, we observed usership graphs in the application showing that a majority of visits and registrations by tutors took place in the final week of the practicum, and not on a more regular basis during the period, as would have been desirable. This may be due to the novelty of the application, but we are inclined to believe that this may also be associated with tutors’ limitations in terms of teaching skills. Tutors must be given training in the fundamentals of formative and continuing evaluation, understanding the latter not only as an isolated action at the conclusion of the practicum rotation, but as a part of the learning process in which continuous feedback is key [20].

According to the students and tutors, one of the limitations of the app is that communication is one-way rather than two-way. This has an impact on the potential for interaction between the student and the clinical tutor. The meta-analysis conducted by Chen et al. (2021) [13] identified better results in terms of learning in studies based on mobile applications with a greater capacity for interaction. We shall therefore work to improve the tool’s communication channels, to allow not only two-way tutor-student interaction, but also multi-directional communication among peer groups. This will facilitate the exchange of experience and learning during clinical practice and will also reinforce the sense of belonging to the group. Nevertheless, as it requires tutors to register from the start of the practicum and enables a connection and direct communication with each student, albeit unidirectional, EVENS may have a positive influence by encouraging tutors to adopt a proactive and committed role.

There were also limitations associated with the numerical imbalance between the intervention and control groups (48 vs. 101), limited participation in the intervention, the small size of some subsamples, and the single-institution setting, which may further limit the generalizability of the findings. Future studies could identify additional academic characteristics related to participation in EVENS, such as previous clinical experience. Sociodemographic and socioeconomic characteristics could also be included to adjust for these potentially relevant covariates.

However, despite these limitations, we consider it necessary to continue gathering knowledge based on teaching experiences that highlight the potential and weaknesses of technologies applied to clinical learning. Future lines of research should address studies with mixed methodologies and a qualitative approach to analyze the views of tutors, teachers, and students on the application of technologies in education from the perspective of critical thinking and reflection. Further studies should include other results and variables associated with the effect of technology on the cognitive burden, critical thinking, technological stress, and interference in the capacity for concentration, as well as a longitudinal approach to ensure long-term effects.

An in-depth analysis of the content of the entries would also be interesting, bearing in mind that their quantity may not be linked to the quality of their content. This analysis could help to identify areas with shortcomings and for improvement in the monitoring and counseling provided during the experiential learning process.

In the future, the integration of artificial intelligence features could enhance the personalization of feedback and the analysis of students’ progression, thereby strengthening the application’s ability to support clinical teaching and learning.

These technologies must be used following a rational and reflective premise, founded on research and pedagogical theories, and applied by duly trained and skilled teachers [21]. In this regard, it is essential that the faculty should receive continuous training in digital skills [22,23].

## 5. Conclusions

The EVENS application did not demonstrate an effect on nursing students’ clinical competence or self-efficacy. However, improvements in these areas were identified in both groups, which were probably associated with the clinical experience gained over time.

EVENS was positively evaluated by tutors regarding its usability and satisfaction, and was perceived as a valuable support tool in their role of training students during clinical placements.

The experience identified areas for improvement within the intervention. In the technical sphere, improvements will be made to the student-tutor and peer group interactivity processes. Regarding conceptual aspects, at the university, we need to continue training tutors, seeking to upgrade their teaching duties and endow them with formative evaluation skills and competence in the use of digital tools. Lastly, a further analysis of the effects of an intervention designed as a mixed longitudinal study is needed.

We hope that this study will contribute to generating knowledge on mobile learning, its potential for implementation, and its effects and limitations in the context of clinical teaching in Nursing.

## Figures and Tables

**Table 1:** Total sample and comparison of mean grades between students who used the application and those who did not.

	Total Sample (*n* = 149)	Intervention Group (*n* = 48)	Control Group (*n* = 101)	*p*
Clinical case report, mean ± S.D.	8.5 ± 1.1	8.5 ± 1.1	8.6 ± 1.1	0.49
Clinical skills labs, mean ± S.D.	8.5 ± 1	8.4 ± 1.0	8.1 ± 1.0	0.21
Follow-up meetings participation, mean ± S.D.	9.5 ± 1	9.5 ± 0.7	9.4 ± 1.1	0.49
Student self-assessment, mean ± S.D.	9.1 ± 0.9	8.9 ± 1.4	9.2 ± 0.6	0.10
Student clinical performance, mean ± S.D.	9.0 ± 0.9	9.0 ± 0.8	9.1 ± 0.9	0.74
Student self-efficacy *, mean ± S.D.	75.2 ± 14.5	73.0 ± 11.1	76.5 ± 16.9	0.60

S.D. = Standard Deviation; Student’s *t*-test for unpaired samples. * The sample sizes in the second evaluation were very small for the total score for the self-efficacy questionnaire (8 in the experimental group and 14 in the control group). Significant *p* ≤ 0.05.

**Table 2:** Comparison of means before and after using the application (*n* = 149).

Components of Student’s Assessment	Experimental Group (n = 48)			Control Group (n = 101)		
	*Pre*	*Post*	*p*	*Pre*	*Post*	*p*
Clinical case report, Mean ± S.D.	7.8 ± 1.3	8.5 ± 1.1	0.01	7.9 ± 1.3	8.6 ± 1.1	<0.001
Clinical skills labs, Mean ± S.D.	8.6 ± 1.0	8.4 ± 1.0	0.37	8.7 ± 1.6	8.5 ± 1.3	0.33
Follow up meetings participation, Mean ± S.D.	9.5 ± 0.8	9.5 ± 0.7	0.99	9.4 ± 0.9	9.4 ± 1.1	0.99
Student self-assessment, Mean ± S.D.	8.9 ± 0.6	8.9 ± 1.5	0.94	8.9 ± 0.7	9.2 ± 0.6	<0.001
Student clinical performance, Mean ± S.D.	9.1 ± 0.8	9.0 ± 0.8	0.85	8.9 ± 0.9	9.0 ± 0.9	0.22
Student self-efficacy *, Mean ± S.D.	73.6 ± 12.7	76.0 ± 11.0	0.79*	74.5 ± 8.9	78.5 ± 15.2	0.21 ^$^

S.D. = Standard Deviation; Student’s *t*-test for related samples * Mean compared in only five pre- and post-data. ^$^ Mean compared in only ten pre- and post-data.

**Table 3:** App usability questionnaire mean scores (*n* = 42).

Item	Mean	SD
1. I think that I would like to use this website often.	3.8	1
2. I found the website simple.	3.7	1.2
3. I thought the website was easy to use.	4	1
4. I think that I would need the support of a technical person to be able to use this website.	3.8	1.1
5. I found the various functions on this website were well integrated.	3.9	0.9
6. I thought there was a lot of consistency in this website.	3.9	1
7. I would imagine that most people would learn to use the website very quickly.	3.8	1.1
8. I found the website very intuitive.	3.9	1
9. I felt very confident using the website.	3.7	1.1
10. I could use the website without having to learn anything new.	3.9	0.9
Total Score	3.8	0.9

SD: Standard Deviation.

## Data Availability

The original contributions presented in this study are included in the article/Supplementary Material. Further inquiries can be directed to the corresponding author.

## Supplementary Materials

The following supporting information can be downloaded at: https://www.mdpi.com/article/10.3390/nursrep16030083/s1, Table S1. Pre-test scores by groups, before using the EVENS application.

## Public Involvement Statement

No public involvement in any aspect of this research.

## Guidelines and Standards Statement

This manuscript was drafted against the TREND Title: TREND Statement Checklist [24] for cuasi-experimental Nonrandomized Evaluations Interventions.

## Abbreviations

The following abbreviations are used in this manuscript:

EVENS	Evaluation Nursing Students
USU	Usability Questionnaire
APP	Application
SD	Standard Deviation
