# Supplementary material for: EVENS (Evaluation Nursing Students): A Mobile Application to Enhance Nursing Students’ Clinical Competence and Self-Efficacy—A Quasi-Experimental Study
Source: Nurs Rep. 2026 Feb 27;16(3):83. doi: 10.3390/nursrep16030083 (PMC13029325; doi:10.3390/nursrep16030083)
Supplement: Supplementary file 1 [file nursrep-16-00083-s001.zip › nursrep-4087702-supplementary.pdf]

Supplementary Table S1. Pre-test scores by groups, before using the EVENS application.

	**Total sample (n=149)**	**Intervention group (*n* = 48)**	**Control group (*n* = 101)**	***p***
Clinical case report, mean ± S.D.	7.9 ± 1.3	7.8 ± 1.3	7.9 ± 1.3	0.62
Clinical skills labs, mean ± S.D.	8.8 ± 0.9	8.6 ± 1.0	8.9 ± 0.9	0.07
Follow up meetings participation, mean ± S.D.	9.4 ± 0.9	9.5 ± 0.8	9.4 ± 0.9	0.40
Student self-assessment, mean ± S.D.	8.9 ± 0.7	8.9 ± 0.6	8.9 ± 0.7	0.79
Student clinical performance, mean ± S.D.	8.9 ± 0.9	9.1 ± 0.8	8.9 ± 0.9	0.28
Student self-efficacy*, mean ± S.D.	74.4 ± 10.0	73.9 ± 9.4	74.6 ± 10.3	0.76

[1] S. D. = Standard Deviation; Student´s T-test for unpaired samples. *The sample sizes were slightly smaller for the total score for the self-efficacy questionnaire (30 in the experimental group and 68 in the control group). Significant $p \leq 0.05$.
